# Theoretical Investigation on Photophysical Properties of Triphenylamine and Coumarin Dyes

**DOI:** 10.3390/ma13214834

**Published:** 2020-10-29

**Authors:** Xinrui Li, Peng Song, Dongpeng Zhao, Yuanzuo Li

**Affiliations:** 1 Department of Physics, Liaoning University, Shenyang 110036, China; lixinrui0320@163.com; 2 College of Science, Northeast Forestry University, Harbin 150040, China; dpzhaonefu@126.com

**Keywords:** optical absorption, excited states, organic molecules, DFT, energy levels

## Abstract

Organic molecules with donor and acceptor configures are widely used in optoelectronic materials. Triphenylamine dyes (TPCTh and TPCRh) are investigated via density functional theory (DFT) and time-dependent DFT. Some microscopic parameters related to light absorption and photoelectric formation are calculated to interpret the experimental performance in dye-sensitized solar cells (DSSC_S_). Considering that coumarin derivatives (Dye 10 and Dye 11) have good donor and acceptor structures, they also have a COOH group used as an anchoring group to connect with semiconductors. Thus, the two dyes’ photophysical and photoelectric properties are analyzed to estimate the performance and application in DSSCs.

## 1. Introduction

After the first report of dye-sensitized solar cells (DSSCs) in 1991 [1], photochemical cells have made a breakthrough and created a new chapter to apply solar energy. For modification of its power conversion efficiency (PCE), the design and synthesis of new dyes exhibit a new channel for improving efficiency in this field. At present, the PCE of DSSCs based on ruthenium reached more than 13% [2,3,4,5]. However, this kind of dye’s manufacturing price is high, and the synthesis method is tedious. Therefore, the development of metal-free and cheap dye sensitizers has become a good idea for many researchers. The dye with excellent performance should meet the following conditions [6,7,8,9,10]: (a) dyes must have a broad absorption range in the visible light region and even part of the infrared region; (b) the dye should be adsorbed well on the TiO_2_ layer and not easy to fall off; (c) molecular lowest unoccupied molecular orbital (LUMO) should be higher than the conduction band (CB) of TiO_2_ so that efficient electron transfer can occur between molecular LUMO and the CB; (d) the highest occupied molecular orbital (HOMO) should be lower than the potential of redox couple to achieve dye regeneration. Therefore, photovoltaic properties are a crucial factor in evaluating DSSCs photoelectric performance. Experimentally, the non-metallic dyes with the D-π-A structure [11,12,13,14] were widely used in the DSSCs, and triphenylamine (TPA) and cyanoacetic acid (CA) have been considered to be the most representative donor and acceptor. Although cyanoacetic acid is usually the preferred representative of the acceptor group, rhodanine-3-acetic acid was also frequently used in DSSCs, because of its easy synthesis and low-cost merits. Different kinds of dyes have been investigated, such as polycyclic benzenoid hydrocarbon [15], triphenylamine [16,17], dimethylaminophenyl and thienyl [18], pyranylidene [19], subphthalocyanines (SubPcs) [20], natural dyes [21] and so on.

The engineering of molecular design provides a meaningful way to improve PCE. For example, Slimi et al. [22] studied the effect of six different acceptor groups on the electron injection ability with indole dithiophene as donor and thiophene as a conjugate bridge. The results show that the 2-(1,1-dicyanomethylene) rhodanine unit has a strong electron absorption ability, which expands the absorption region of the absorption spectra and increases the absorption intensity. A series of quinoline based dyes within the π- spacers of cyanovinyl and thiophene have been reported by P. Pounraj et al. [23]. The results show that the coumarin based and N-hexyltetrahydroquinoline donors are more suitable for DSSC application. Arooj et al. [24] used a computer-aided rational design (CARD) method to illustrate how to improve organic dye optical properties, and this method can simulate and develop more effective organic chromophores. Sang-aroon et al. [25] studied the photovoltaic performance of monascus dyes in DSSCs, and the results showed that monascus dyes, including monascin, rubropunctatin and rubropunctamine, would be better photosensitizers in DSSC. Some specific factors affecting the PCE have been reported by Xu et al. [26], finding that appropriate electron injection driving force and regenerative driving force are beneficial to molecular photoelectric performance. Besides them, the effects of single dye, co-sensitization and series structure on device efficiency are efficient ways to change PCE [27].

Recently, two non-metallic dyes (TPCTh and TPCRh) were synthesized by Hemavathi et al. [28]. The effect of acceptor structures of cyanoacetic acid and rhodamine-3-acetic acid on molecular photoelectric performance has been investigated. Here, the ground state and excited state properties of TPCTh and TPCRh in vacuum and solvent were studied to explain the effect of difference on performance. The frontier molecular orbital (FMO), absorption spectra and electron injection driving force of organic dyes were studied by DFT and TD-DFT theory [29,30,31,32], respectively. Considering the experimental research by Martins et al. on fluorescent probe molecules [33], one studied these two molecules 10 and 11 with donor and acceptor (–COOH) for the potential utility of DSSCs. To further predict the PCE of coumarin dyes 10 and 11 in DSSCs, one simulates their photoelectric properties, which provides help for better analysis of the relationship between molecular structure and molecular photoelectric properties.

## 2. Methods

The DFT [34] and Lee-Yang-Parr correlation function (B3LYP) [35] are used to obtain geometry structure and excited-state performance of investigated molecules at basis set 6-31 G (d) level. All the calculations will not be affected by all symmetrical tubes. Based on the molecular structure and design scheme, the molecular excitation energy and oscillator compressive strength are calculated in the framework of TD-DFT [36]/Cam-B3LYP [37]/6-31 G (d) level. In light of the effectiveness of organic solvents in the excited state’s optimal control, the PCM was selected to calculate the excitation energy [38], and methylene chloride was selected as the organic solvent [28,33]. To check the reliability of the calculation method, we adopt B3PW91/6-31G (d), BPV86/6-31G (d), MPW1PW91/6-31G (d), HSEH1PBE/6-31G (d), and PBEPBE/6-31G (d) for the functional correction (see Supporting materials). Besides, dye with larger hyperpolarizability have increased their utility in organic optoelectronic materials [39,40], and the first hyperpolarizability of the total static data is obtained by the following formula [39]:

(1) $$\beta_{tot}=\sqrt{\beta_{x}^{2}+\beta_{y}^{2}+\beta_{z}^{2}}$$

A single static component of the equation can be calculated as follows,

(2) $$\beta_{l}=\beta_{lll}+\frac{1}{3}\underset{l\neq m}{\sum }\left(\beta_{lmm}+\beta_{mlm}+\beta_{mml}\right)$$

where $\beta_{lmn}\left(l,m,n=x,y,z\right)$ are tensor components of hyperpolarizability. The total hyperpolarizability can be obtained as follows:

(3) $$\beta_{tot}={\left[{\left(\beta_{xxx}+\beta_{xyy}+\beta_{xzz}\right)}^{2}+{\left(\beta_{yyy}+\beta_{yzz}+\beta_{yxx}\right)}^{2}+{\left(\beta_{zzz}+\beta_{zxx}+\beta_{zyy}\right)}^{2}\right]}^{1/2}$$

All calculations are carried out using the Gaussian 09 program (Gaussian 09 revision A.1.; Gaussian Inc.: Wallingford, CT, USA) [41].

## 3. Results and Discussion

### 3.1. Geometry

**Figure 1:**
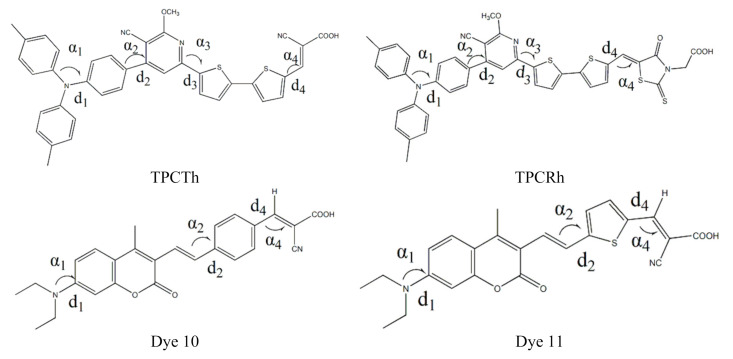
Chemical structures of the four original molecules Triphenylamine dyes (TPCTh, TPCRh), 10 and 11.

The molecular chemical bond lengths and dihedral angles are given in Table 1. The molecular geometry structures of four dyes (TPCTh, TPCRh, Dye 10 and Dye 11) were investigated in vacuum dichloromethane solvent by DFT/ B3LYP/6-31G(d) without symmetry constraints. The chemical structure and the atomic number of the studied molecules are shown in Figure 1, and four dyes both have donor and acceptor units (the carboxylic acid acts as an adsorption). Table 1 shows molecular chemical bond lengths and dihedral angles. For molecules TPCTh and TPCRh, the angles between donors under vacuum and solvent were 30.74°, 29.55° and 32.51°, 29.90°, and this means that the molecular structure of the donor part is twisted. The distorted donor structure can inhibit molecular aggregation at the semiconductor surface [42,43,44]. For molecular TPCTh, the angles of D_1_-D_2_ (α_2_), D_2_-π (α_3_) and π-A (α_4_) are 42.39°, −0.97° and −0.53°, respectively. The corresponding bond lengths were 1.480 Å, 1.459 Å and 1.425 Å; for TPCRh, the dihedral angles are 42.96°, 0.58° and −178.79°, respectively. The corresponding bond lengths were found to be 1.480 Å, 1.458 Å and 1.430 Å. In the solvent, the angles of D_1_-D_2_ (α_2_), D_2_-π(α_3_) and π-A (α_4_) for TPCTh are 43.02°, −0.89° and 0.10°, respectively. The corresponding bond lengths were 1.478 Å, 1.458 Å and 1.421 Å, respectively; for TPCRh, the dihedral angles are 43.02°, 0.05° and −179.57°, and the corresponding bond length are 1.478 Å, 1.457 Å and 1.427 Å, respectively. Thus, the donor part shows a sizeable twisted structure to reduce the self-aggregation of molecules, and π conjugation bridges and partial receptor aberrations were significantly reduced, and the π-A part of the TPCTh tends to the plane structure. This will make it easier to transfer the charge through the π bridge to the receptor part, and this phenomenon is more evident in the solvent. Table 1 indicates that, under the same conditions, there was no significant change in the molecular bond length of the rest, and the binding length of the cyanoacetic acid receptor was slightly reduced than that of the rhodamine-3-acetic acid receptor.

For the two molecules’ dihedral angles, in the vacuum, the twist angles of donor groups were 15.29° for Dye 10 and 14.25° for Dye 11, respectively. This part of the donor structure has a reduced twisted angle compared with triphenylamine dyes. Besides, the primary molecular skeletons for both dyes almost form a planar configuration. According to the corresponding molecular bond length, the bond length of molecule 11 is shorter, which is more conducive to charge transfer.

### 3.2. Frontier Molecular Orbital

**Figure 2:**
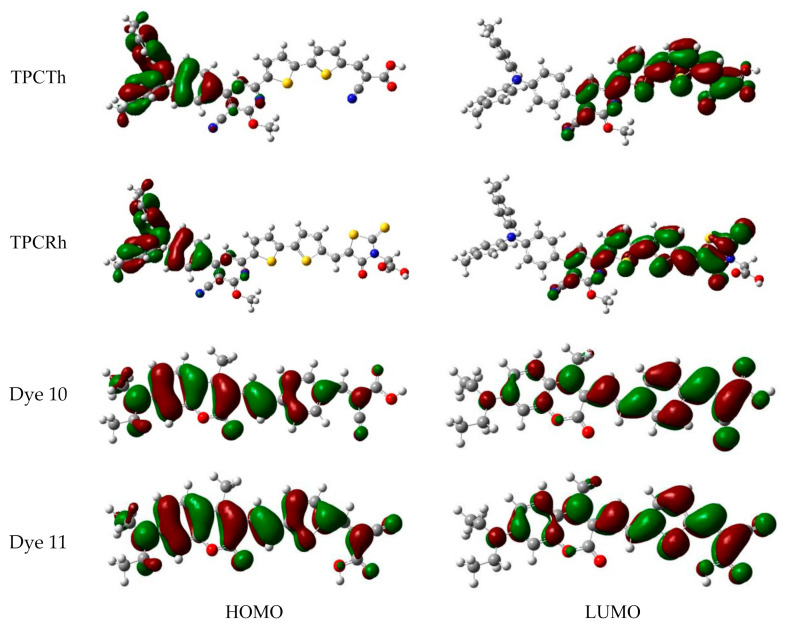
Frontier molecular orbital (FMO) diagrams of four original molecules.

FMO was usually utilized to reflect molecular and excitation transition characteristics [45,46,47]. The energy levels of four molecules (TPCTh and TPCRh, 10 and 11) simulated in vacuum and solvent are shown in Table 2. The corresponding FMO graphs in the vacuum are presented in Figure 2. The electron cloud of HOMO orbit belongs to the π type, and the electron cloud of LUMO orbit belongs to the π* type. In the vacuum phase, the electron cloud density of the HOMO orbital of TPCTh is concentrated in the partial delocalization of the TPA donor, and the HOMO energy value is −5.13 eV. The LUMO orbital is mainly focused on the receptor region. A few of them exist on the π conjugated bridge’s right side, and the LUMO energy value is −2.97 eV. Therefore, when electrons are excited and jumped from the HOMO to LUMO orbital, the electrons are transferred from the molecular donor part to the conjugated bridge and receptor part, which leads to effective intramolecular charge transfer (ICT). The electron cloud density of the HOMO orbit of TPCRh is mainly concentrated in the donor part, and the HOMO energy value is −5.12 eV. The LUMO orbital is distributed in the region between the conjugated bridge and acceptor, and the LUMO energy value is −2.95 eV. The two dyes’ molecular orbital energy levels and energy gaps are very close (see Table 2), and TPCTh has a carboxyl group in the anchoring group. LUMO extends to cyanoacetic acid that is suitable for charge transfer. In contrast, TPCRh exhibits less LUMO charge density (Figure 2), leading to weak electron coupling of dye and semiconductor. In the solvent, HOMO and LUMO’s energy levels are lower than that in a vacuum, and thus the energy gap has decreased trend.

As shown, the HOMO orbit of coumarin molecule 10 is distributed on the whole molecular orbital, and the energy level is −5.27 eV; the LUMO is distributed on the right half of the π conjugated bridge, the acceptor and the donor, with the value of −2.58 eV; HOMO and LUMO of molecular 11 have similar electron cloud distribution as molecule 10, which energy corresponding to −5.29 eV for H and −2.70 eV for L, respectively. The order of energy gap between the two molecules is 10 (2.69 eV) > 11 (2.59 eV), respectively. The comparison between the two dyes and semiconductors shows that the two molecules can achieve effective charge separation and electron injection. The overall performance of the molecule 11 tends to be more planar, resulting in a smaller energy gap. This effect is better observed in the solvent due to dye molecules’ interaction with the solvent, which results in a red-shifted absorption spectrum.

**Figure 3:**
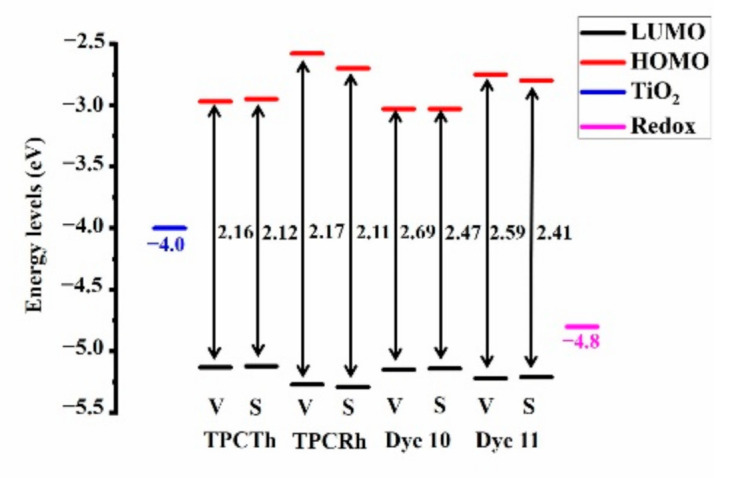
Energy-level diagram of four original molecules.

Figure 3 shows the molecular energy level diagram, including dyes and semiconductors, and dye sensitizers are higher than the CB (−4.0 eV) surface, ensuring the effective injection of electrons. Compared with experimental molecule TPCTh and TPCRh, Dye 10 and Dye 11 have a higher LUMO energy level, which is very beneficial for electron injection. Meanwhile, these dyes’ HOMO energy level is lower than that of redox potential I¯/I_3_¯electrolyte (−4.8 eV). Thus, these electron-lost sensitizers can receive electrons from the redox couple. Notably, the HOMO energy values of TPCTh and TPCRh are significantly higher than those of molecules 10 and 11, which can indicate that Dyes 10 and 11 are comfortable to obtain electrons and regenerate dyes. From the viewpoint of energy levels of Dyes 10 and 11, they have the potential possibility to use in DSSC_S_ because of the energy-level matching.

### 3.3. Absorption Spectra

**Figure 4:**
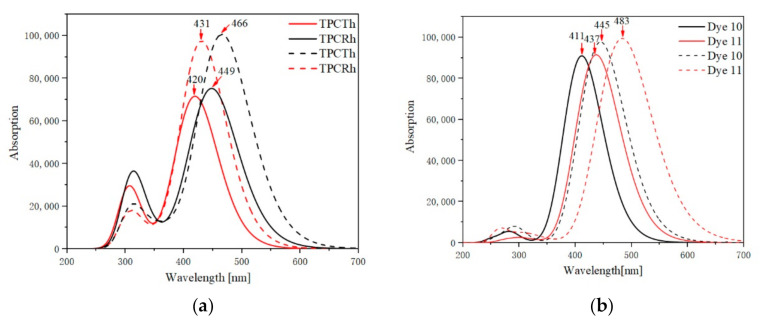
(**a**) The absorption spectra of molecules TPCTh and TPCRh in vacuum (solid lines) and solvent (dotted lines). (**b**) The absorption spectra of the molecules 10 and 11 in vacuum (solid lines) and solvent (dotted lines).

TD-DFT of the quantum chemical method is widely utilized in the investigation of molecular absorption and emission characteristics. The transition properties of the dyes were obtained via the TD-DFT/CAM-B3LYP/6-31G (d) based on ground state geometry [48,49,50]. The functional correction was carried out by using B3PW91/6-31G (d), BPV86/6-31G (d), MPW1PW91/6-31G (d), HSEH1PBE/6-31G (d) and PBEPBE/6-31G (d) (see Supporting Materials Table S1). The calculated oscillator strength (*f*), the excitation energy (*E*), the maximum absorption wavelength (*λ_max_*) and the main electron transition are shown in Table 3, and Figure 4 shows the molecular absorption spectra of four dyes in the vacuum and solvent.

Table 3 shows that, in the vacuum, the absorption peaks of molecular TPCTh and TPCRh are 419.93 nm and 431.15 nm, respectively. The oscillator strengths are 1.5747 and 2.1580, respectively. Two dyes are 448.63 nm and 465.67 nm in the solvent condition, which is in accordance with the experimental value [28]. The functional correction by using B3PW91/6-31G (d), BPV86/6-31G (d), MPW1PW91/6-31G (d), HSEH1PBE/6-31G (d) and PBEPBE/6-31G (d) shows more massive red-shifted results. From Table 3, it can be found that both dyes can perform efficient photoelectric conversion in the visible region. The calculated spectra parameters of molecules 10 and 11 are shown in Table 4, indicating that the molecular excitation state occupies the dominant transition from HOMO to LUMO. Molecular absorption peaks of 10 and 11 are 411.32 nm and 436.94 nm in a vacuum; however, in the solvent, both dyes showed a redshift of about 33.41 nm and 46.33 nm, respectively. Compared with TPCTh, Dye 11 showed redshift absorption about 17 nm, and corresponding oscillator strength is higher than TPCTh, which means that Dye 11 possesses a widen absorption range, and it may be beneficial to increase solar utilization.

### 3.4. Hyperpolarizability

The hyperpolarization of the molecular structure describes the molecular system’s positive response to the external electrostatic field, reflecting ICT strength and the nonlinear optical characteristics of the system. To scientifically study the relationship between structure and the nonlinear optical properties, the hyperpolarizability of two molecules was investigated. Like DSSC, the migration of electronics from donors to acceptors and semiconductors is beneficial to the electric current generation. Because the hyperpolarizability *β* is proportional to the dipole moment difference and transition dipole moment and inversely proportional to *E_eg_*, the estimation of hyperpolarizability could provide an understanding of the ICT ability. The hyperpolarizability can be calculated as follows [51]:

(4) $$\beta \propto \frac{\Delta \mu_{eg}{\left(\mu_{eg}\right)}^{2}}{E_{eg}^{2}}$$

The calculated results are listed in Table 5. It can be seen that the *β_xxx_* position of TPCTh is positive, indicating that the electron distribution in the molecule is close to the nuclear charge, while the value of TPCRh is negative. Then, the *β_tot_* of TPCTh is the largest. Combined with the oscillator strength and transition energy (*E_eg_*) of the two molecules, it can be predicted that TPCTh has a massive dipolar moment difference (Δ*μ_eg_*), thus enhancing the dye intramolecular charge transfer performance. Compared with TPCTh, Dyes 10 and 11 have a larger value of *β_tot_*, dipolar moment difference between the two molecules becomes larger will help improve the intramolecular charge transfer performance of dyes.

### 3.5. Driving Force of Electron Injection

To better predict the PCE of dye, light-harvesting efficiency (LHE) and electron injection driving force (Δ*G_inject_*) are obtained and listed in Table 6. LHE has a close relationship with oscillator strength; that is to say, the higher oscillator strength contributes to light absorption, and another factor affecting the *Jsc* is the (Δ*G_inject_*), which can be obtained from follows [52]:

(5) $$\Delta G_{inject}=E_{\mathrm{ox}}^{{\mathrm{dye}}^{*}}-E_{\mathrm{CB}}$$

Among them, $E_{\mathrm{ox}}^{{\mathrm{dye}}^{*}}$ and $E_{\mathrm{CB}}$ are the redox potential energy of excited state and the CB of TiO_2_(4.0 eV), respectively. $E_{\mathrm{ox}}^{{\mathrm{dye}}^{*}}$ was calculated by [53]:

(6) $$E_{\mathrm{ox}}^{{\mathrm{dye}}^{*}}=E_{\mathrm{ox}}^{\mathrm{dye}}-\Delta E$$

Among them,$E_{\mathrm{ox}}^{\mathrm{dye}}$ and ΔE are the redox potential energy and electron transition energy, respectively, and the ground state redox potential energy is calculated by HOMO energy. The values of Δ*G_inject_* for four dyes are negative so that the spontaneous process of electron injection can occur. As shown in Table 6, the absolute value of Δ*G_inject_* is calculated to be TPCTh > TPCRh > Dye 10 > Dye 11 in vacuum condition, and they can satisfy the requirement of electron driving, which indicates that the excited dyes promote the electrons injection from the sensitizer to the TiO_2_ conduction band. However, excessive injection values Δ*G_inject_* has a show of produce electron accumulation and energy redundancy in semiconductors and exerts a negative influence of *Voc*. After the dye loses the electron, it needs to get the electron from the electrolyte and return to the original state. One calculates four dyes’ regeneration ability based on dye and electrolyte [54]. The Δ*G_reg_* values of the four investigated dyes are shown in Table 6. It is important to have enough driving force for dye regeneration since it impacts the electron recombination process (from semiconductors to excited dyes) and further reduces the electron loss. We can find that Dyes 10 and 11 are found to be a larger regeneration driving force(*∆G_reg_*), thus enhancing their ability to obtain electrons instinctively compared to that of the first two dyes. Consequently, Dyes 10 and 11 show the potential to be appropriate due to the well Δ*G_reg_*.

### 3.6. Chemical Parameters

Ionization potential (IP) and electron affinity (EA) [55] can describe the potential barrier of holes and electrons. The IP, EA and chemical reaction parameters are calculated as listed in Table 7. From Table 7, TPCTh and TPCRh more easily accept electron because of larger EA, and Dyes 10 and 11 can lose electron owing to their lower energy of losing electrons. It seems that for the same donor and conjugated bridge dyes (TPCTh and TPCRh), the different acceptor (cyanoacetic acid /rhodanine-3-acetic) have a minor effect on IP and EA; while for Dyes 10 and 11, the replacement by benzene ring and thiophene ring in conjugated bridge make IP and EA changed by 0.01 eV. Chemical hardness h, electrophilicity *W* and electron-accepting *W+* or electron-donating power *W-* are calculated based on IP and EA for the sake of explaining the nature of dyes and pigments [56]. As shown in Table 7, chemical hardness *h* of TPCTh and TPCRh is lower than that of Dyes 10 and 11, and electrophilicity *W* is higher than the two fluorescent molecules. Thus TPCTh and TPCRh show a lower impedance during charge redistribution. One also used Koopman’s theorem to calculate chemical descriptors (*h*, *W*, *W+* and *W−*) employing HOMO and LUMO levels (results are listed in Supporting materials Table S2), finding similar trend for *h* and *W* obtained by IP and EA. This means that the energy gap is directly related to chemical hardness; that is to say, the larger energy gaps lead to larger *h*. *W+* and *W-* power values by calculation with IP and EA show the difference of about 0.1 eV between triphenylamine and coumarin dyes, and coumarin dyes have the larger *W+* and lower *W−* that indicate well electron-donating and accepting ability. The two parameters calculated with Koopman’s theorem demonstrated the same trend (see Supporting Materials Table S2).

### 3.7. Fluorescent Lifetime

Fluorescent lifetime (*τ*) formula could be used to assess an electron’s state properties, and a long fluorescent lifetime means an increment of the electron injection probability into TiO_2_. It is an essential factor to study the excited state electron transfer process and to evaluate molecular photoelectric performances, which can be calculated by equation [57]:

(7) $$\tau =\frac{2\pi \varepsilon_{0}m_{e}\hbar^{2}c^{3}}{e^{4}E^{2}f}$$

where *c* is light speed, *f* and *E* represent oscillator strength and energy in the fluorescent state; *e*, *m_e_*, *ε_0_* and *ħ* stands for the elementary charge, electron mass, vacuum permittivity and reduced Planck constant. As shown in Table 8, in vacuum, the calculated *τ* of TPCTh and TPCRh are 2.23 ns and 1.71 ns, respectively, and molecules 10 and 11 are 1.50 ns and 1.70 ns, respectively. We found that the range of four dyes in vacuum is 1.50 ns–2.23 ns. Even in the solvent, the change of fluorescence lifetime is not significant. Some studies have also shown that the electron injection time is generally on the picosecond or femtosecond time scale, while the fluorescence lifetime of the dye studied is on the nanosecond time scale [58]. Hence, it can be found that these dyes can ensure that electrons can be transferred to the conduction band of semiconductors to achieve an effective charge transfer process.

## 4. Conclusions

This work theoretically investigated the TPCTh and TPCRh using DFT and TD-DFT methods. The simulated spectra are in good accordance with experimental values. TPCTh molecule shows well conjugation that is conducive to ICT and higher PCE performance in TPA dyes, which can be contributed to the larger values of Δ*G_inject_*, *τ* and *β_tot_*. Besides, the electron density of molecular orbital TPCTh extends to cyanoacetic acid that is suitable for charge transfer and increases electron coupling with semiconductor, showing that TPCTh containing cyanoacetic acid receptor is more ideal for DSSCs. Meanwhile, calculation on fluorescence molecules 10 and 11 (containing donor and acceptor structures COOH) show higher LUMO and lower HOMO than TPA molecules, which can effectively conduct electron injection and electron regeneration. Molecule 11 also shows larger redshift absorption and stronger oscillator strength. Combined with *β_tot_*, LHE and other parameters, two kinds of fluorescent dyes may be promising to improve the photophysical properties of dyes and have a fair application in DSSCs.

## Figures and Tables

**Table 1:** Specific bond length (Å) and dihedral angle (°) in vacuum and CH_2_Cl_2_.

Condition	Dye	α_1_	α_2_	α_3_	α_4_	d_1_	d_2_	d_3_	d_4_
Vacuum	TPCTh	30.74	42.39	−0.97	−0.53	1.408	1.480	1.459	1.425
	TPCRh	32.51	42.96	0.58	−178.79	1.408	1.480	1.458	1.430
	Dye 10	15.29	0.52	-	−179.81	1.383	1.458	-	1.445
	Dye 11	14.25	−0.44	-	−0.02	1.382	1.442	-	1.428
Solvent	TPCTh	29.55	43.02	−0.89	0.10	1.405	1.478	1.458	1.421
	TPCRh	29.90	43.02	0.05	−179.57	1.405	1.478	1.457	1.427
	Dye 10	11.10	−1.27	-	179.92	1.373	1.457	-	1.441
	Dye 11	10.24	−0.24	-	−0.17	1.371	1.440	-	1.418

**Table 2:** Energy levels and energy gaps of four molecules in vacuum and solvent (eV).

Condition	Dye	HOMO	LUMO	Δ_H-L_
Vacuum	TPCTh	−5.13	−2.97	2.16
	TPCRh	−5.12	−2.95	2.17
	Dye 10	−5.27	−2.58	2.69
	Dye 11	−5.29	−2.70	2.59
Solvent	TPCTh	−5.15	−3.03	2.12
	TPCRh	−5.14	−3.03	2.11
	Dye 10	−5.22	−2.75	2.47
	Dye 11	−5.21	−2.80	2.41

**Table 3:** Transition energies (eV), absorption peaks (nm) and oscillator strengths of TPCTh and TPCRh in vacuum and solvent.

Condition	Dye	State	*E* (eV)	*λ_max_* (nm) *Cal/Exp* *	CI main	*f*
Vacuum	TPCTh	S1	2.9525	419.93	(0.66501)H-1→L	1.5747
		S2	3.3830	366.49	(0.59391)H→L	0.0705
		S3	3.9898	310.76	(0.37870)H→L+1	0.3016
		S4	4.0729	304.41	(0.46327)H-1→L+1	0.3540
		S5	4.3876	282.58	(0.45742)H-2→L	0.0050
		S6	4.4368	279.44	(0.63913)H→L+4	0.0204
	TPCRh	S1	2.8757	431.15	(0.66421)H-1→L	2.1580
		S2	3.2007	387.36	(0.48232)H-3→L	0.0001
		S3	3.4209	362.43	(0.55261)H→L	0.1171
		S4	3.8288	323.82	(0.52941)H-1→L+1	0.0490
		S5	4.0114	309.08	(0.36677)H→L	0.3288
		S6	4.2232	293.58	(0.50253)H-2→L	0.0359
Solvent	TPCTh	S1	2.7636	448.63/440 *	(0.66980)H-1→L	1.6597
		S2	3.3011	375.59	(0.57628)H→L	0.1291
		S3	3.8786	319.66	(0.50145)H-1→L+1	0.3237
		S4	3.9939	310.43	(0.37873)H→L+1	0.4943
		S5	4.3345	286.04	(0.50054)H-8→L	0.0129
		S6	4.3596	284.39	(0.41447)H-2→L	0.0147
	TPCRh	S1	2.6625	465.67/475 *	(0.66557)H-1→L	2.2336
		S2	3.3059	375.04	(0.49065)H-4→L	0.0002
		S3	3.3336	371.92	(0.52607)H→L	0.1848
		S4	3.6453	340.12	(0.54339)H-1→L+1	0.0672
		S5	3.9605	313.05	(0.39766)H→L	0.3772
		S6	4.1553	298.37	(0.49634)H-2→L	0.0676

* Experimental results from Ref [28].

**Table 4:** Transition energies (eV), absorption peaks (nm) and oscillator strengths of the two molecules in vacuum and solvent.

Condition	Dye	State	*E* (eV)	*λ_max_* (nm) *Cal/Exp* *	CI main	*f*
Vacuum	Dye 10	S1	3.0143	411.32	(0.65466)H→L	2.0236
		S2	3.9795	311.56	(0.56046)H→L+1	0.0038
		S3	4.3609	284.31	(0.52120)H-1→L	0.0936
		S4	4.4513	278.54	(0.60998)H-4→L	0.0199
		S5	4.5024	275.37	(0.40443)H-2→L	0.0042
		S6	4.9201	252.00	(0.35361)H-1→L+1	0.0458
	Dye 11	S1	2.8376	436.94	(0.67606)H→L	2.0357
		S2	3.8590	321.29	(0.51796)H→L+1	0.0237
		S3	4.1581	298.18	(0.49775)H-1→L	0.0358
		S4	4.4399	279.25	(0.40334)H-2→L	0.0035
		S5	4.5545	272.23	(0.53340)H-4→L	0.0222
		S6	4.7732	259.75	(0.56177)H-6→L	0.0000
Solvent	Dye 10	S1	2.7879	444.73/452 *	(0.63828)H→L	2.1753
		S2	3.7448	331.08	(0.56737)H→L+1	0.0029
		S3	4.2091	294.56	(0.50979)H-1→L	01467
		S4	4.3891	282.48	(0.63416)H-4→L	0.0259
		S5	4.4622	277.85	(0.40533)H-2→L	0.0055
		S6	4.7638	260.26	(0.41197)H-1→L	0.0505
	Dye 11	S1	2.5655	483.27/483 *	(0.66357)H→L	2.2135
		S2	3.6205	342.45	(0.51009)H→L+1	0.0365
		S3	4.0221	308.26	(0.47227)H-1→L	0.0847
		S4	4.3773	283.24	(0.39392)H-2→L	0.0043
		S5	4.4294	279.91	(0.53716)H-4→L	0.0306
		S6	4.6905	264.33	(0.35719)H→L+1	0.1337

* Experimental results from Ref [33].

**Table 5:** Hyperpolarizabilities of TPCTh, TPCRh, Dye 10 and 11(a.u).

Dye	*β_xxx_*	*β_xyy_*	*β_xzz_*	*β_yyy_*	*β_yzz_*	*β_yxx_*	*β_zzz_*	*β_zxx_*	*β_zyy_*	*β_tot_*
TPCTh	35,764	−5537	−349	309	−2885	404	−133	−526	27	29,963
TPCRh	−34,535	10,465	627	48	−4372	682	−404	221	93	23,724
Dye 10	45,798	−2403	−614	27	133	−23	14	−71	−17	42,782
Dye 11	−33,101	2738	−53	−200	34	22	16	74	1	30,416

**Table 6:** Electronic properties of four dyes in vacuum and solvent, respectively (eV).

Condition	Dye	Δ*G_inject_*	$E_{\mathrm{ox}}^{\mathrm{dye}}\;$	$E_{\mathrm{ox}}^{{\mathrm{dye}}^{*}}\;$	Δ*G_reg_*	LHE	Stokes Shift (nm)
Vacuum	TPCTh	−1.82	5.13	2.18	0.33	0.973	57
	TPCRh	−1.76	5.12	2.24	0.32	0.993	63
	Dye 10	−1.74	5.27	2.26	0.47	0.994	51
	Dye 11	−1.55	5.29	2.45	0.49	0.991	52
Solvent	TPCTh	−1.61	5.15	2.39	0.35	0.978	80
	TPCRh	−1.52	5.14	2.48	0.34	0.994	85
	Dye 10	−1.57	5.22	2.43	0.42	0.993	91
	Dye 11	−1.36	5.21	2.64	0.41	0.994	88

**Table 7:** Ionization potential (IP) (electron affinity (EA)) and chemical parameters for four dyes.

Dye	IP	EA	*h*	*W* (eV)	*W+*	*W−*
TPCTh	5.039	3.173	0.933	4.517	1.698	7.098
TPCRh	5.035	3.169	0.933	4.510	1.695	7.086
Dye 10	5.014	2.992	1.011	3.961	1.768	6.047
Dye 11	4.996	3.058	0.969	4.186	1.716	6.479

**Table 8:** The fluorescent lifetime (ns) of TPCTh, TPCRh, dyes 10 and 11.

Condition	Dye	*τ* (ns)
Vacuum	TPCTh	2.23
	TPCRh	1.71
	Dye 10	1.50
	Dye 11	1.70
Solvent	TPCTh	2.56
	TPCRh	2.03
	Dye 10	1.81
	Dye 11	2.08

## Supplementary Materials

The following are available online at https://www.mdpi.com/1996-1944/13/21/4834/s1, Table S1. Calculated transition energy and oscillator strength in S1 of the TPCTh, TPCRh, Dye 10, Dye11 in B3PW91/6-31G(d), BPV86/6-31G (d) MPW1PW91/6-31G(d), HSEH1PBE/6-31G (d), PBEPBE/6-31G (d) by TD-DFT method in solvent results.; Table S2. Chemical parameters for four dyes with Koopman’s theorem.
