# Supplementary material for: Theoretical Investigation on Photophysical Properties of Triphenylamine and Coumarin Dyes
Source: Materials (Basel). 2020 Oct 29;13(21):4834. doi: 10.3390/ma13214834 (PMC7663121; doi:10.3390/ma13214834)
Supplement: Supplementary file 1 [file materials-13-04834-s001.pdf]

Table S1. Calculated transition energy and oscillator strength in S1 of the TPCTh, TPCRh, Dye 10, Dye11 in B3PW91/6-31G(d), BPV86/6-31G (d) MPW1PW91/6-31G(d), HSEH1PBE/6-31G (d), PBEPBE/6-31G (d) by TD-DFT method in solvent results.

Dye	Excitation State Parameters	B3PW91 6-31G(d)	BPV86 6-31G (d)	MPW1PW91 6-31G (d)	HSEH1PBE 6-31G (d)	PBEPBE 6-31G (d)
	E (eV)	1.85	1.04	2.07	1.70	1.04
TPCTh	Strength *f*	0.1231	0.0699	0.1686	0.0889	0.0690
	λ_{max} (nm)	668.75	1188.69	600.18	728.71	1186.43
	E (eV)	1.88	1.03	2.09	1.71	1.03
TPCRh	Strength *f*	0.1493	0.0631	0.2513	0.0905	0.0622
	λ_{max} (nm)	661.22	1198.07	592.71	726.96	1198.96
	E (eV)	2.23	1.74	2.34	2.19	1.74
Dye 10	Strength *f*	1.5210	0.9247	1.6767	1.4252	0.3483
	λ_{max} (nm)	556.69	713.65	529.97	565.81	711.00
	E (eV)	2.20	1.83	2.22	2.18	1.83
Dye 11	Strength *f*	1.8168	1.1397	1.9429	1.7513	1.1390
	λ_{max} (nm)	564.19	677.18	544.83	568.11	678.24

Table S2. Chemical parameters for four dyes with Koopman's theorem.

Dye	*h*	*W*	*W+*	*W-*
**TPCTh-s**	0.933	4.517	1.698	7.098
**TPCRh-s**	0.933	4.510	1.695	7.086
**Dye 10-s**	1.011	3.961	1.768	6.047
**Dye 11-s**	0.969	4.186	1.716	6.479
**TPCTh-s**	1.058	3.949	1.883	5.986
**TPCRh-s**	1.058	3.943	1.881	5.976
**Dye 10-s**	1.236	3.206	2.077	4.576
**Dye11-s**	1.203	3.328	2.045	4.806
